# Molecular subtypes as a prognostic breast cancer factor in women users of the São Paulo public health system, Brazil

**DOI:** 10.1590/1980-549720230028

**Published:** 2023-05-29

**Authors:** Stela Verzinhasse Peres, Paola Engelmann Arantes, Marcela de Araújo Fagundes, Alexandre Muxfeldt Ab’Saber, Daniel Luiz Gimenes, Maria Paula Curado, René Aloisio da Costa Vieira

**Affiliations:** Fundação Oncocentro de São Paulo, Department of Information and Epidemiology – São Paulo (SP), Brazil.; A.C. Camargo Cancer Center, Centro International de Pesquisa, Cancer Epidemiology and Statistics Group – São Paulo (SP), Brazil.; Fundação Oncocentro de São Paulo, Department of Pathology – São Paulo (SP), Brazil.; Universidade de São Paulo, Clinical Hospital – São Paulo (SP), Brazil.; Grupo Oncoclínicas de São Paulo, Department of Mastology – São Paulo (SP), Brazil.; Universidade de São Paulo, Faculty of Medicine of Botucatu, Graduate Program in Obstetrics and Gynecology – Botucatu (SP), Brazil.; Hospital do Câncer de Barretos, Graduate Program in Oncology – Barretos (SP), Brazil.

**Keywords:** Breast neoplasms, Survival rate, Biomarkers, Social vulnerability, Neoplasias da mama, Taxa de sobrevida, Biomarcadores, Vulnerabilidade social

## Abstract

**Objective:**

This study aimed to analyze the prognosis of women with breast cancer by molecular subtypes, sociodemographic variables, and clinical and treatment characteristics.

**Methods:**

This hospital-based retrospective cohort study analyzed 1,654 women over 18 years of age diagnosed with invasive breast cancer from 2000 to 2018. Data were extracted from Brazil’s Oncocenter Foundation of São Paulo. The variables analyzed were age, histology, molecular subtypes, clinical staging, treatment type, and diagnosis-to-treatment time. Cox regression analysis was applied to estimate death risk.

**Results:**

Women with HER-2-positive (nonluminal) and triple-negative molecular subtypes were more than twice more likely to be at risk of death, with adjusted hazard ratio — HR_adj_=2.30 (95% confidence interval — 95%CI 1.34–3.94) and HR_adj_=2.51 (95%CI 1.61–3.92), respectively. A delayed treatment associated with an advanced clinical stage at diagnosis increased fourfold the risk of death (HRadj=4.20 (95%CI 2.36–7.49).

**Conclusion:**

In summary, besides that interaction between advanced clinical stage and longer time between diagnosis and treatment, HER-2-positive (nonluminal) and triple-negative phenotypes were associated with a worse prognosis. Therefore, actions to reduce barriers in diagnosis and treatment can provide better outcome, even in aggressive phenotypes.

## RESUMO

**Objetivo:**

O objetivo deste estudo foi analisar o prognóstico de mulheres com câncer de mama de acordo com os subtipos moleculares, variáveis sociodemográficas, características clínicas e de tratamento.

**Métodos:**

Este foi um estudo de coorte retrospectivo de base hospitalar. Foram analisadas 1.654 mulheres maiores de 18 anos diagnosticadas com câncer de mama invasivo entre 2000 a 2018. Os dados foram extraídos da Fundação Oncocentro de São Paulo, Brasil. As variáveis analisadas foram idade, histologia, subtipo moleculares, estadiamento clínico, tipo de tratamento e tempo entre o diagnóstico e tratamento. A análise de regressão de Cox foi aplicada para estimar o risco de morte.

**Resultados:**

As mulheres que apresentaram os subtipos moleculares HER-2-positivo (não luminal) e triplo negativo tiveram risco de morte quase duas vezes maior respectivamente, com razão de risco ajustada — HR_aj_=2,30 (intervalo de confiança de 95% — 95%IC 1,34–3,94) e HR_aj_=2,51 (95%IC 1,61–3,92). O atraso no tratamento associado ao avanço do estadiamento clínico ao diagnóstico aumentou em quatro vezes o risco de morte (HR_aj_=4,20 (IC95% 2,36–7,49).

**Conclusão:**

Os fenótipos HER-2-positivo (*não luminal*) e triplo negativo, além da interação entre estágio clínico avançado e maior tempo entre o diagnóstico e o tratamento, associaram-se a pior prognóstico. Assim, ações para reduzir as barreiras no diagnóstico e tratamento podem proporcionar melhores resultados, mesmo em fenótipos agressivos.

## INTRODUCTION

Breast cancer is the most frequent malignant neoplasm in women worldwide. A total of 2,261,419 million new cases and 684,996 thousand related deaths were estimated in 2020^1^. In Brazil, 66,280 new breast cancer cases in women are expected for each year of the 2020–2022 triennium, corresponding to an estimated risk of 6,161 new cases per 100 thousand women. In 2019, 18,068 women died from breast cancer, representing 16.4% of all cancer-related deaths in women^2^. According to the CONCORD-3 Study, the highest 5-year relative survival rates among women with breast cancer between 2010 and 2014 were observed in the United States (90.2%) and New Zealand (91.8%). The survival rates in countries such as India and Thailand are lower, approximately 67%, while the relative survival rate is 75.2% in Brazil^3^.

The main prognostic factors of women’s breast cancer include age at diagnosis, clinical stage, histological classification, treatment options, and molecular subtype^4,5,6,7^. After the discovery of HER-2, breast cancer was subdivided into the luminal A and B subtypes, the HER-2+ subtype, and the triple-negative subtype, changing the clinical practice. The triple-negative phenotype, which affects 15–20% of women with breast cancer, is associated with the worst prognosis and survival^7,8,9,10,11^.

Breast cancer has been analyzed within the classic variables tumor, node, metastasis — TNM (AJCC 8th edition), histology, and hormone receptor (HR) status, which classifies high- and low-risk patients for adjuvant treatment. This new approach, observing distinct characteristics of the molecular subtypes concerning age, ethnicity, radiological presentation, response to therapy, and distant metastasis type, has identified patients who are candidates for specific therapies with positive effects on survival rates^12,13,14^. Although the molecular analysis of the risk of recurrence in some molecular subtypes is included in the latest American Joint Committee on Cancer (AJCC) guidelines, the molecular subtype still needs to be incorporated into the TNM staging approach^15,16^.

However, limited Brazilian studies have evaluated the link between the molecular subtype’s classification and prognoses in breast cancer patients, due to the higher cost of these tests, mainly for the public health system^7,17^. Therefore, this study aimed to analyze prognosis factors in women with invasive breast cancer treated in the public health system in the state of São Paulo, according to sociodemographic, clinical and molecular subtypes.

## METHODS

In this retrospective cohort study, we collected data on a hospital-based cancer registry of 1,654 women aged 18 years or above diagnosed with invasive breast cancer in the public health system, from 2000 to 2018. We selected breast cancer using the International Classification of Diseases, Oncology, 3_rd_ edition (ICD-O-3), topographical codes C50.0 to C50.9, and morphological codes 8050/3, 8211/3, 8480/3, 850_/3, 851_/3, 852_/3, 853_/3, and 854_/3.

The data were obtained from São Paulo’s hospital-based cancer registry (RHC/SP) and the Immunohistochemistry Laboratory database at the Oncocenter Foundation of São Paulo (FOSP). Only patients who had not received prior treatment were eligible. Patients with incomplete or missing data regarding biomarkers estrogen receptor (ER) progesterone receptor (PR), HER-2, Ki-67 and clinical staging (CS) at diagnosis and previous treatment were excluded (Supplementary Figure 1).

### Immunohistochemical analysis

Formalin-fixed paraffin-embedded (FFPB) scrolls, with both positive and negative controls, were subjected to automated immunohistochemical examination (Tissue Microarray Assay, TMA), with antigenic recovery PTLink (Dako^®^) and incubation, retrieval and counterstaining in AutoStainer Link 48 (Dako^®^), using a susceptible polymer with FLEX “ready-to-use” antibodies. Samples with at least 1% estrogen- or progesterone-positive tumor nuclei were considered positive. The colored cell proportion score (PS) applied ranges from 0 to 5, and the coloring intensity (IS) score ranges from 0 to 3. The scoring system used was the Allred Score (AS), calculated by the sum of the PS and IS scores. HER-2 positivity was defined as a score of 3+ (complete and strong staining observed in over 30% of tumor cells). A fluorescence in situ hybridization (FISH) test was performed on tumors with a score of 2+ (moderate staining of the membrane, observed in more than 10% of tumor cells). The tumor was considered positive when the receptor was overexpressed in the amplification analysis^18^.

For Ki-67, the samples were subjected to immunohistochemical techniques using the streptavidin-biotin-peroxidase method. Sections of 3 μm thickness were obtained from samples fixed in formalin and embedded in paraffin, which were placed on glass slides labeled with 3-aminopropyltriethoxysilane (Sigma-Aldrich, St. Lois, Missouri, USA). After that, the cuts were deparaffinized and rehydrated, followed by immersion in a 2% H_2_O_2_/methanol solution to block endogenous peroxidase. Antigenic retrieval was performed in a water bath for 45 minutes with a 0.01 M citrate buffer solution (pH 6.0) at 96°C (Merck, Darmstadt, Hessen, Germany). Samples were incubated with the Ki-67 primary antibody. The LSAB Kit (Dako Corporation, Carpinteira, USA) was used to apply the biotinylated secondary antibody, followed by incubation with the streptavidin-peroxidase complex. Diaminobenzidine chromogen (DAB) (Biocare, Concord, CA, USA) was used to reveal the reaction, and the slides were counterstained with Harris hematoxylin and mounted with Entellan (Merck, Germany). After each step, the slides were washed in phosphate-buffered saline for 5 minutes. Positive controls for all antibodies were included. Negative control was performed by omitting the primary antibody^19^.

The probabilistic linkage was applied to the data obtained from the immunohistochemistry laboratory’s information systems and the RHC/SP to obtain a single database containing demographic, clinical, and laboratory data, both located at the FOSP, using the Open RecLink III program, version 3.1.615. The technique used was based on research with similar databases that have shown sensitivity (90%) and specificity for breast cancer data (99.0%)^20^. The variables used in the matching process were the patient’s name and date of birth. The Soundex codes of the first name (PBLOCO) and last name (UBLOCO) were used for blocking. Confirmatory variables used to determine a true match were, when available, the mother’s name, home address, public health system (SUS) card number, or another personal identification document. In addition, the first diagnosed tumor was chosen in women with more than one tumor, and the most aggressive histological tumor type was selected in bilateral cases.

### Covariates

The database with the following analyzed variables was obtained: age at diagnosis, education level, histology (invasive ductal, invasive lobular, others), tumor size — T1/T2 (≤ 5 centimeters) and T3/T4 (> 5 centimeters) — and lymph nodes — N0, N1/N2 and N3 (TNM 7^th^ edition), CS at diagnosis, the time between diagnosis and treatment, and treatment types: 1) chemotherapy (CTX); 2) surgery + CTX; surgery + radiotherapy (RXT) + CTX; 3) surgery + RXT + CTX + hormone therapy (HT); and 4) other combinations. The molecular subtypes were classified according to the St. Gallen Consensus^14^: luminal A, luminal B (HER-2 negative), luminal B (HER-2 positive), HER-2 positive (nonluminal), and triple-negative. 

The Research Ethics Committee of the Dr. Mário de Moraes Altenfelder Silva Municipal Maternity-School Hospital authorized this study under protocol no. 4.106.934.

### Statistical analysis

We performed descriptive data analysis for all patients (n=1,654) through absolute and relative frequencies, central tendency, and dispersion measures. The overall survival (OS) rate was performed over five years on patients diagnosed until December 31, 2013 (n=894). Survival time was determined by the date of diagnosis and the vital status (dead or alive) at the date of the last follow-up. The date of death was confirmed through an active search to determine the vital status of patients using the Registration System for Users of the Unified Health System (CADSUS) platform and passively via linkage with the State Statistical Data Analysis System Foundation (FSEADE), responsible for registering deaths in the state of São Paulo, and updating information through follow-up with the participants’ physicians. The Kaplan-Meier limit product estimator test was applied to calculate the five-year survival probability, and the log-rank test was applied to compare the curves. Cox univariate and multiple regression analyses were used to estimate the hazard ratio (HR) and its respective 95% confidence interval (95%CI). 

For multiple modeling, variables with significant statistical values and p<0.20 were selected. A stepwise technique was used, testing the lowest to the highest p-value. The final model was built with the following assumptions:

1. No change in HR >10%;
2. Improved accuracy by 95%CI;
3. Total degrees of freedom allowed for the outcome; and
4. Interaction effect between covariates.

Control for the confounding covariates age, year of diagnosis and treatment were considered adjustments^21^.

The Schoenfeld residual scale was applied to test the proportional hazard. The data were analyzed using the STATA software version 14.

## RESULTS

Three hundred and seventy-four (22.6%) of the 1,654 women with invasive breast cancer treated by the Brazilian public health system died. The mean age was 56.8 (standard deviation — SD=13.2), with a median of 56, ranging from 22 to 96 years. Of these, 48% completed High School, and 55.7% showed a time between diagnosis and treatment above two months. The histological classification was 71.9% for invasive ductal carcinoma (Table 1). Regarding the biomarkers, 25.9% of the tumors were negative for estrogen receptors, 33.9% were negative for progesterone receptors, 85.5% were negative for HER-2, and 68.3% had a Ki-67 level ≥14%. Regarding the molecular subtypes, the luminal B/HER-2- phenotype was the most frequent (41.2%) (Table 2).

**Table 1:** Demographic and clinical characteristics of patients diagnosed with invasive breast cancer in the public health system of Sao Paulo, Brazil, Cancer Registry Center, Oncocenter Foundation of São Paulo, 2000–2018.

Variables	Categories	n	%
Age	<40	149	9.0
	40 to 49	380	23.0
	50 to 69	837	50.6
	>69	288	14.4
Education	Illiterate/Middle school incomplete	437	37.3
	Middle school complete	370	31.6
	High School/Graduated	365	31.3
	Missing	482	29.1
Histology	Invasive ductal	1,189	71.9
	Invasive ductal/lobular	201	12.2
	Invasive lobular	60	3.6
	Others	204	12.3
T Tumor	T1/T2	1,106	68.1
	T3/T4	517	31.9
Lymph nodes	N0	742	44.9
	N1/N2	741	44.8
	N3	52	3.1
	Distant Metastasis	119	7.2
Clinical staging at diagnosis	I	288	17.4
	II	757	45.8
	III	490	29.6
	IV	119	7.2
Total		1,654	100

**Table 2:** Biomarkers, molecular subtypes of patients diagnosed with invasive breast cancer in the public health system of Sao Paulo, Brazil, Cancer Registry Center, Oncocenter Foundation of São Paulo, 2000–2018.

Variables	Categories	n	%
Treatment	Chemotherapy (CTX)	231	14
	Surgery/CTX	224	13.5
	Surgery/RTX/CTX	251	15.2
	Surgery/RTX/CTX/HT	652	39.4
	Other combinations	296	17.9
Time between diagnosis and treatment	<2 months	349	44.3
	2–4 months	236	29.9
	≥4 months	203	25.8
Estrogen	Positive	1,226	74.1
	Negative	428	25.9
Progesterone	Positive	1,093	66.1
	Negative	561	33.9
HER-2	Positive	240	14.5
	Negative	1,414	85.5
Ki-67*	<14%	523	31.7
	≥14%	1,127	68.3
Molecular subtypes	Luminal A	480	29
	Luminal B (HER-2 negative)	682	41.2
	Luminal B (HER-2 positive)	105	6.3
	HER-2 positive (*nonluminal*)	135	8.2
	Triple-negative	252	15.2
Total		1,654	100

*4 patients with ignored values.

For the 5-year OS, 894 women with invasive breast cancer were analyzed. The median follow-up time was 54.13 months, ranging from 0.3 to 60 months. In general, the 5-year OS was 72.5% (95%CI 69.2–75.5%), with the losses to follow-up at 6.5%. The 5-year OS rate was lower in women >69 years of age, advanced CS at diagnosis, and patients with negative tumors of estrogen and progesterone receptor. In the univariate regression analysis, the risk of death among patients with negative ER and PR was HR=2.45 (95%CI 1.86–3.21) and HR=2.51 (95%CI 1.91–3.28), respectively. For those with HER-2-positive tumors and Ki-67 levels ≥14%, the risk of death was lower [HR=1.65 (95%CI 1.25–2.19)] than that of women with HER-2-negative tumors and Ki-67 levels <14% [HR=2.12 (95%CI 1.55–2.91)]. The worst 5-year OS rates were observed in patients with positive HER-2 (negative) (nonluminal) (55% [HR=3.58; 95%CI 2.21–5.78]) and triple-negative tumors (50% [HR=3.44; 95%CI 2.32–5.10)]. The univariate analysis did not reveal that the time between diagnosis and treatment was a risk factor for death (Supplementary Table 1).

**Figure 1.**
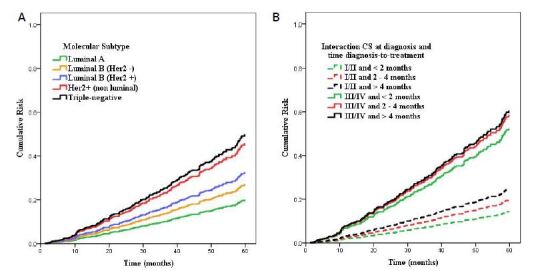
Hazard function to molecular subtype (A) and interaction between clinical staging at diagnosis and diagnosis-to-treatment time (B), adjusted by age, year of diagnosis and treatment in patients diagnosed with invasive breast cancer in the public health system of São Paulo, Brazil. Cancer Registry Center, Oncocenter Foundation of São Paulo, 2000–2013.

In Table 3, the independent prognostic factors for the risk of death were molecular subtypes, the interaction between CS at diagnosis and time between diagnosis and treatment, adjusted for age at diagnosis, year of diagnosis and treatment. Women with molecular subtypes HER-2-positive tumors (nonluminal) and triple-negative were more than twice at risk of death, HR_adj_=2.30 (95%CI 1.34–3.94) and HR_adj_=2.51 (95%CI 1.61–3.92), respectively. Likewise, the delayed treatment associated with advancing CS at diagnosis increased three to fourfold the risk of death (p<0.001). Although the Schoenfeld test rejects the hypothesis of proportionality of risk (p=0.022), it is possible to identify the linearity between the categories of molecular subtypes (A) and the interaction between CS at diagnosis and time between diagnosis and treatment (B) during follow-up (Figure 1).

**Table 3:** Prognostic factors for the risk of death in women with invasive breast cancer. Cancer Registry Center, Oncocenter Foundation of São Paulo, 2000–2013.

Variable	Category	HR_adj_*	95%CI		p
			Lower	Upper	
Molecular subtypes	Luminal A	Ref			
	Luminal B (HER-2 negative)	1.35	0.91	2.03	0.141
	Luminal B (HER-2 positive)	1.63	0.79	3.40	0.189
	HER-2 positive (*nonluminal*)	2.30	1.33	3.94	0.003
	Triple-negative	2.51	1.61	3.92	<0.001
Interaction CS at diagnosis and Time between diagnosis and treatment (months)	CS I/II and <2	Ref			
	CS I/II and 2 a 4	1.37	0.76	2.46	0.297
	CS I/II and ≥4	1.71	0.97	3.03	0.064
	CS III/IV and <2	3.63	2.21	5.98	<0.001
	CS III/IV and 2 a 4	4.06	2.28	7.22	<0.001
	CS III/IV and ≥4	4.20	2.36	7.49	<0.001

CS: clinical staging. *adjusted by age, year of diagnosis and treatment.

## DISCUSSION

In this study, which constitutes the largest Brazilian study in patients treated exclusively by the public health system, molecular subtype was determined to be an independent risk factor, as was the delayed treatment associated with advancing CS at diagnosis to the risk of death in women with breast cancer treated at public health service.

We found that the distribution of the molecular subtypes in this study was similar to that observed nationally and internationally^5,10,22,23^. In Brazil, Carvalho et al.^24^. evaluated the molecular subtypes of invasive breast tumors distributed in the country’s five regions and showed a distribution of 27.7% luminal A, 37.1% luminal B, 10.4% luminal B HER-2+, 8.9% HER-2+, and 10.4% triple-negative. In the Southeast region, 28.8% of tumors were luminal A, 39.5% were luminal B, 9.7% were triple-positive, 7.9% were HER-2, and 14.0% were triple-negative. In the AMAZONA study with women with stages I-III tumors exclusively, 49.4% of the tumors were luminal A, 8.7% were luminal B/ negative HER-2, 13.2% were luminal B/ positive HER-2, 7.7% were HER-2+, and 21.0% were triple-negative^7^.

The 5-year OS rate in this study was similar to that of the other Brazilian studies and studies of populations in low-income countries (72.5%)^11,22^, when compared to high-income European countries, the United States, Canada and China^3,22,25,26^. We should highlight that the patients in this study were treated in the public health system, where diagnoses are often delayed and the time between diagnosis and the first treatment is prolonged (approximately 85 days)^27^. Our study reported a poor OS rate in patients with more than four months between diagnosis and treatment. An important fact is that 55.7% of the women started treatment after four months, far from the recommended by Brazilian law. Furthermore, the educational level is less than nine years of schooling, low in this population, where 31.3% have High School or Graduate level. 

The 5-year OS was superior in those with luminal A and B subtype tumors, 75 to 90%. When stratifying breast cancer patients from the public health system by molecular subtype, the 5-year OS rates were lower in patients with HER-2 positive (nonluminal) and triple-negative tumors independently of clinical stage. However, the lowest 5-year OS were in women with the triple-negative, which agrees with other studies^5,10,17,22,23^. Regarding the women from the public health system in this study, less than 10% of patients were under 40, similar to the other countries^7,24,28^. Several researchers have shown that breast cancer in patients <40 years have a lower OS. This unfavorable prognosis is usually associated with advanced staging, HER-2 overexpression, and nodal metastasis. Concerning tumor differentiation, lymphatic involvement, necrosis, and negative ER were observed^29^.

Most patients were between 50 and 69 years old, reinforcing the importance of screening in this age group^2^. The lower 5-year OS rate in women ≥70 years can be explained by low life expectancy and comorbidities^30^. Other studies have shown that worse survival rates are only observed in elderly patients with triple-negative tumors. The relationship between age and the triple-negative subtype is unclear in younger women^31,32^.

In this study, the 5-year OS rate of patients with clinical stage IV of the disease was 28.2%, similar to the study by Marque et al. in the Brazilian Northwest. In Brazil, new targeted therapies became available in SUS in 2013. An increase in the OS rate of patients with clinical stage IV tumors, young patients, and those with different molecular subtypes has been observed^33^. Recent studies have revealed that advances in treatment, compared with advances in screening, were associated with more pronounced reductions in overall breast cancer mortality rates and improved survival rates between 2000 and 2012^33,34,35,36^.

Our results showed that there is a dose-response gradient associated with the molecular subtypes; that is, the greater the tumor’s aggressiveness, the greater the risk of death. Adjusting for clinical stage and proposed treatment revealed that triple-negative breast cancer patients had the worst prognosis over five years of follow-up. In HER-2-positive and triple-negative tumors, the complete pathological response rate can reach 60% or higher, which is related to the longer survival time of these patients, as adjuvant hormonal therapy is indicated mainly for positive hormonal receptor luminal tumors in menopausal women^23,37^. We should highlight that luminal B/positive HER-2 tumors have shown similar survival outcomes to luminal B/negative HER-2 tumors in some studies, which may have been influenced by the development of anti-HER-2 therapy^38,39^. However, not all HER-2-positive patients in this study received anti-HER-2 therapy, as it only became available to patients in the public health system in 2013 and is only administered to those with metastatic disease^40^.

This study observed that the longer the time between diagnosis and treatment, regardless of molecular subtype, the worse the OS indices. Even so, this effect is not isolated and is only identified in advanced staging at diagnosis. However, the risk of death can be three to four times higher. According to Brazilian data, the longer the time between diagnosis and treatment, the shorter the patient’s survival. Rapid intervention is essential for treatment effectiveness in the more advanced stages^41^. Social vulnerability can have a profound impact on a patient’s prognosis. Hence survival factors, such as low education level and lack of immediate access to diagnosis and treatment, must be better evaluated to identify the existent barriers^42^.

This research has limitations. The analyzed data were extracted from the RHC to describe the sociodemographic and clinical treatment adopted. Nevertheless, with the discovery of biomarkers to guide the treatment, this data has yet to be available in the public database, which precludes more accurate studies. However, this study identified women with a complete set of biomarkers to classify all cases at RHC, analyzing the profile of women with breast cancer in the Brazilian public health system. There is a need to incorporate the new types of therapies available as essential variables. For example, trastuzumab was made available to public health system patients in 2013, and a double blockade was never performed for HER-2 patients. Furthermore, the survival time may be influenced by the type of medication available to public health system patients. 

The molecular subtype is an independent risk factor for death among the Brazilian women cohort in the public health system depending on several epidemiological characteristics; those with HER-2-positive (nonluminal) and triple-negative tumors have the worst prognoses. These findings will facilitate the comparison of prognostic factors and epidemiological characteristics between heterogeneous populations, such as Brazilian and Latin American women, with other populations.
